# 

**Published:** 1846-03

**Authors:** 


					) Tol II.
M1KCH, 1816.
No. I* '
AMERICAN
JOUR?A?
tnial Scitnii.
Wm? w?l!R m
QUARTERLY
PUBLISHED UNDER THE AUSPICES Of TBS
m SocCets of Bental Surgeons.
editors:
CHAPIN A. HARRIS M. D., D. D. S.
AMOS WESTCOTT, M. D., D.D.S.
EDWARD MAYNARD, M. D., D.D.S.
ARMSTRONG fc BERRY
JOHN W. WOODS, PRINTER.
'
9 5 OO per annum* payable In advance.

				

